# Site fidelity, size, and morphology may differ by tidal position for an intertidal fish, *Bathygobius cocosensis* (Perciformes-Gobiidae), in Eastern Australia

**DOI:** 10.7717/peerj.2263

**Published:** 2016-07-28

**Authors:** Lucie A. Malard, Katrina McGuigan, Cynthia Riginos

**Affiliations:** School of Biological Sciences, University of Queensland, St Lucia, QLD, Australia

**Keywords:** Environmental gradient, Goby, Intertidal ecology, Morphometrics, Survival, Mark-recapture

## Abstract

The intertidal zone is a transitional environment that undergoes daily environmental fluctuations as tides rise and fall. Relatively few fish species are adapted to endure the physiological pressures of this environment. This study focused on *Bathygobius cocosensis* (Gobiidae), a common intertidal fish in New South Wales, Australia. We investigated whether shore height impacted site fidelity, survival probability, fish size, and morphological traits with respect to tidal height. Mark-recapture methods were used over a five month period to determine if individuals in high shore pools had greater site fidelity; fish in high tide pools were more than twice as likely to be recaptured in their original pool than fish from low tide pools. High pool individuals were, on average, smaller with larger eyes and longer snouts relative to their size as compared to low pool individuals. We discuss several mechanisms that could cause the observed pattern in morphological variation. Ultimately, this study suggests that within species behaviour and morphology differ by tidal position for an intertidal fish.

## Introduction

Many ecological studies have been conducted in the intertidal environment (Dakin, Benneti & Pope, 1948; Connell, 1972; Paine, 1974; Menge, 1976; Thomson & Lehner, 1976), as this environment’s highly transitional physicochemical properties over small spatial distances facilitate the study of abiotic effects on biota (Connell, 1972; Daniel & Boyden, 1975; Morris & Taylor, 1983). The intertidal zonation concept was introduced in the early 1900’s and describes the distribution of invertebrates in narrow bands, in accordance to the period of air exposure from low to high tide (Klugh, 1924; Dakin, Benneti & Pope, 1948; Connell, 1972; Paine, 1974; Somero, 2002; Chappuis et al., 2014). Vertical distributions of fishes living in the intertidal are frequently described in terms of life histories (Gibson, 1972; Yoshiyama, Sassaman & Lea, 1986; Griffiths, West & Davis, 2003; Cox et al., 2011; White, Hose & Brown, 2015). Resident species spend their full adult life cycle in the intertidal (typically following a pelagic larval stage) and are well adapted to endure high physiological stress. They are found across the entire intertidal and are usually the most abundant species in the high intertidal zone (Thomson & Lehner, 1976; Griffiths, West & Davis, 2003; Cox et al., 2011). Opportunists, or secondary residents, usually spend one stage of their life cycle (often juveniles) in rockpools, and transient species are usually pelagic species accidentally caught with the ebbing tide and are poorly adapted to intertidal life (Zander, Nieder & Martin, 1999; Griffiths, 2003b; Griffiths, West & Davis, 2003). Thus, opportunist and transient species are most often found in the lower intertidal zone where interspecific competition is strong and predation by other fishes can be high (Thomson & Lehner, 1976; Menge & Sutherland, 1987; Griffiths, West & Davis, 2003). Although resident species may experience extreme gradients of abiotic and biotic conditions, particularly those that covary with shoreline height, few studies have quantified intraspecific behavioral or morphological variation within resident fishes with regards to shoreline height.

Site fidelity and homing behaviours are common among intertidal resident fishes and describe the ability of an adult individual to return to a formerly occupied microhabitat location such as an individual rockpool (Yoshiyama et al., 1992; Griffiths, 2003a; White & Brown, 2013). Site fidelity and homing abilities are thought to be essential for survival, helping individuals avoid stranding in unsuitable pools (Gibson, 1972; Griffiths, 2003a; White & Brown, 2013), which could be especially important in thermally stressful habitats such as the high intertidal. Because submersion time in the high intertidal is short, time for fish to move from pool to pool is short as compared to the low intertidal, which is completely submerged for most of the tidal cycle. Despite several studies of site fidelity in intertidal fishes (Yoshiyama et al., 1992; Griffiths, 2003a; White & Brown, 2013), none have accounted for shoreline height, which, in light of the strong habitat differences, could impact site fidelity and survival.

Fish size is ecologically important, as predation vulnerability, foraging, breeding success, and resistance to stress are influenced by an individual’s size (Webb, 1984; Magnhagen & Kvarnemo, 1989; Martin, Horn & Chotkowski, 1999; Scharf, Juanes & Rountree, 2000). Within species, uneven size distributions across the intertidal zone have been reported, with smaller individuals typically, but not always, more prevalent in the high intertidal zone, and larger individuals more common in the lower intertidal (Zander, Nieder & Martin, 1999; Hernández et al., 2002; Polgar & Bartolino, 2010; Cox et al., 2011). Small individuals might be excluded from the low intertidal zone due to competition and predation (Pauly, 1979; Hernández et al., 2002; White, Hose & Brown, 2015). Variation in physiological tolerances with body size has also been reported for intertidal fishes and might contribute to variation in size across the intertidal zone (Pauly, 1979; Hernández et al., 2002; White, Hose & Brown, 2015). This present study will determine if size zonation occurs within a species of Gobiidae on the Australian east coast.

If resident fishes have strong site fidelity then morphological matching to specific microhabitats should be advantageous. There is extensive literature on how fish body shape relates to feeding behaviour (Keast, 1978; Wimberger, 1992; Mérigoux & Ponton, 1998), swimming performance (Webb, 1984; Scharf, Juanes & Rountree, 2000), and fitness (Sato, 2006). In the context of resident intertidal fish, we expect thermal stress, food availability, predation pressures, and intraspecific competition to differ with respect to shoreline height, which could be reflected in morphological differences. There are several examples of morphological matching for intertidal invertebrates that are associated with specific microhabitats and predators, including: distinct size, shape, and shell patterning in periwinkles (reviewed by Johannesson et al., 2010); size and carapace patterning in crabs (Todd et al., 2006); and predator-induced changes to barnacle test shape (Lively, 1986). However, whether there are consistent differences in shape (or size) within intertidal fish species on small scales is unknown. Morphological variation across the shoreline could arise due to three, not mutually exclusive, mechanisms. First, phenotypic plasticity, where environment induces expression of a particular phenotype (e.g., Day, Pritchard & Schluter, 1994). Second, via habitat choice, where individuals with different morphologies choose different habitats (e.g., Bolnick et al., 2009), which would include cases where morphologically distinct individuals from different life history stages occupy different habitats. Finally, via selection (e.g., Hendry et al., 2006), where individuals surviving in high tide pools have different morphology to those surviving in low tide pools. Regardless of the mechanism, the initial question, and the one we aim to address at present, is whether adult fish exhibit morphological differences by shoreline microhabitat.

Here, we test whether resident intertidal fish show behavioural and morphological differences according to shoreline height. We focus on *Bathygobius cocosensis*, a permanent resident of intertidal pools of exposed rocky shores, from the most extreme high intertidal to low shallow subtidal (Griffiths, 2003b; White, Hose & Brown, 2015), throughout the Indo-Western Pacific region (Sale, 1991; Myers, 1999). Like many gobies, *B. cocosensis* feed on small benthic invertebrates (Griffiths, 2003a; Cox et al., 2011) and are well adapted to endure physiological stress as they can withstand both extreme temperature and salinity fluctuations of high shoreline pools (Griffiths, 2003a; White, Hose & Brown, 2015). *B. cocosensis* have a planktonic larval duration of approximately 30 days based on otolith growth rings (J Thia, 2016, unpublished data). At the conclusion of their larval stage, *B. cocosensis* recruit to the intertidal and we have observed fresh recruits (less than 10 mm total length, <10 days post settlement; J Thia, 2016, unpublished data) widely distributed across the intertidal (with recruitment highest from February through March at the study site of Hastings Point). Although adults have high site fidelity and homing capacity (Griffiths, 2003a; White & Brown, 2013), whether or not individuals recruit to a specific microhabitat and remain there is not known.

We predicted that *B. cocosensis* living in high and low rockpools would experience different ecological conditions and that site fidelity, survival, and morphology may differ as a consequence. Site fidelity and survival probability were measured using a mark-recapture approach. We anticipated that site fidelity would be greater among high pool individuals to avoid stranding in unsuitable pools. Morphometrics were used to test whether biotic and abiotic differences between high and low shoreline pools were associated with variation in size and body shape between habitats.

## Material & Methods

### Sampling sites

All field work was conducted at Hastings Point, NSW, Australia (−28.36, 153.58) from September 2014 through February 2015. Sampling was conducted in a 150 m zone parallel to the shoreline, which experiences a tidal range of 1.6 m. Random numbers generated by R v3.1.1 (R Core Team, 2014) were attributed to locations determined on Google Earth v7.1. Once on site, discrete rockpools (tide pools) were chosen by walking a straight line from the random point location to the water line. Sampled rockpools had to be at least 10 cm deep with a minimum volume of 40 L as pilot field observations suggested that pools outside of these characteristics were often devoid of fish. We then assigned pools to one of two categories, using “high” and “low” shoreline position. High pools were exposed during every low tide and for most of the tidal cycle. Low pools were only exposed at spring tides and marginally during neap tides when they received intermittent wave splashes. Designated high and pools differ markedly (and consistently) in their biotic characteristics. High pools are largely devoid of macroalgae and above the barnacle zone, whereas low pools maintain extensive macroalgal cover through all seasons and are often contain *Pyura stolonifera* ascidians (signature species of the low intertidal/shallow subtidal for the region). Similar criteria have been used in other studies (for example, Cox et al., 2011; White, Hose & Brown, 2015) and our categories are consistent with the pool definitions used by White, Hose & Brown (2015) in more southern locations of NSW. (Photographs of tide pool locations and tidepools can be found in the Supplemental information.) As reported for designated high and low shoreline pools in other localities (e.g., Cox et al., 2011; White, Hose & Brown, 2015) fish communities are also quite distinct. There was greater fish species richness in low pools, whereas in high pools fishes consisted mainly of *Bathygobius cocosensis* and occasionally juvenile *Microcanthus strigatus* (L Malard, 2015, unpublished data).

Four pools, two high and two low, were selected for this study, based on the aforementioned criteria (shore height and algal cover and sufficient depth and volume). Selected pools were more than 20 m apart to minimize the probability of tagged fish moving between studied tide pools. Previous studies showed that *B. cocosensis* exhibit strong homing abilities, with 20% of individuals returning to their original pools of capture from translocated distances as great as 20 m (Griffiths, 2003a).

For each sampling event, all fish were captured by completely emptying the pool using a battery-powered bilge pump. While the pool was empty, fish were flushed from crevices using a wash bottle and captured using hand nets to ensure the full sampling of the pool (Griffiths, 2003a; Griffiths, West & Davis, 2003). We made every attempt to capture all fishes from the drained pools and only stopped once a pool was completely drained and five minutes of searching (particularly through macroalgae) without finding any more fish had elapsed. Fishes other than the target species were held in a 20 L container of seawater and were released to their original pool after it was refilled. *B. cocosensis* individuals were held in a separate container for the mark recapture experiment (see below) and then returned to their home pool.

### Site fidelity and survival probability

On the first visit to the site (September 2014), all *B. cocosensis* captured from each of the four target pools were marked to investigate whether site fidelity and survival differed among high shore and low shore rockpools. Because seasonal recruitment typically ceases by July, all fish encountered in September were juveniles and not fresh recruits; thus, our analyses (tagging and morphometrics) are entirely based on juvenile and adult fish (>18 mm total length), not new recruits. Fish were anaesthetized (0.3 × 10^−3^ mg L^-1^ of Aqui-S^®^) and tagged on their side with Visible Implant Elastomer (VIE) subdermal tags (Northwest Marine Technologies^®^, Inc.). VIE tags are biocompatible fluorescent elastomers injected sub-dermally as a liquid that solidifies after injection (Catalano et al., 2001; FitzGerald, Sheehan & Kocik, 2004). Fish were allowed to recover for at least 10 min in aerated seawater before being returned to the pool from which they were captured. This process was repeated five times, in September, October, November 2014 and January and February 2015. During each sampling event, a different colour of VIE dye was used, in order to identify each cohort and to recognize the previous time of marking for recaptured individuals. As the studied pools were over 20 m apart, migration of marked individuals between sampling pools would be unlikely, and an individual tagging scheme would be difficult to implement given the size of the fish and animal welfare concerns (i.e., an individual marking scheme would necessitate multiple injections per fish per capture event). Newly caught fish were tagged for the first time on their left side. Recaptured individuals were tagged again (on the right side with the date-specific dye) and previous tags were used as indicators of when they were first sampled.

In mark-recapture experiments such as this one, an individual can disappear because they lose their tag, because they die, or because they leave the sampling region. VIE tags do not detectably impact growth and mortality rates (FitzGerald, Sheehan & Kocik, 2004) and typically have high retention rates over 6 months (Catalano et al., 2001; Olsen & Vøllestad, 2001; Josephson et al., 2008). Of particular relevance here, a previous study of tag retention and mortality in *B. cocosensis* demonstrated high tag retention and low mortality of VIE tagged fish (for White & Brown, 2013: 100% retention and 0% mortality for 42 days, and for Griffiths (2002): 77 ± 19% tag retention and 7% mortality compared to 11% mortality in a non tagged, control group after 90 days). Tag loss introduces measurement error into mark–recapture analyses, but here we have no reason to expect tag loss to occur differentially between high and low shoreline pools such that this source of error would bias data interpretation. We did not directly observe any mortality events in this study, either as fish deaths during capture, VIE tagging, or during photography. Thus, although we expect some fish to have died between monthly sampling events, we assume that these mortalities are independent of handling effects. Untagged fish were detected during monthly sampling events; they were presumed to be immigrants and were subsequently tagged.

Given our repeat sampling design, the Cormack–Jolly–Seber (CJS) (Cormack, 1964; Jolly, 1965; Seber, 1965) model is appropriate for estimating survival and site fidelity (White & Burnham, 1999; Cooch & White, 2006). CJS models are based on the main assumption that marked and unmarked individuals have the same capture probability. This method also uses instances where tagged individuals reappear after being absent for one or more sampling events to separately estimate survival and emigration. The CJS method was implemented in MARK v8 (White & Burnham, 1999) and was used to estimate the survival (*ϕ*) and encounter probabilities (*p*) (Cooch & White, 2006). An encounter history, which recorded encounters of individuals with regards to sampling occasion and pool ID, was input into MARK and the resultant models estimated the survival (*ϕ*) probability between each recapture event and encounter probabilities (*p*) at each event. Both parameters can be either constant (⋅), time-dependent (*t*), have a group effect (*g*, in this case high vs. low rockpool) or have a group and time-dependent effect (*g*^∗^*t*). (Notation as per MARK instruction and literature, Cooch & White (2006)). All sixteen possible models were tested, from the simplest model *ϕ*(.)*p*(.) to the most general with the most parameters *ϕ*(*g*^∗^*t*)*p*(*g*^∗^*t*). From these sixteen possible models, the most likely model was selected using the corrected Akaike Information Criterion (AIC_*c*_) (Burnham & Anderson, 2002). The model with the lowest AIC_*c*_ was considered the best model and models with a ΔAIC_*c*_ >2 were not considered further. The goodness-of-fit (GOF) was tested using the median $\hat {c}$ function (Cooch & White, 2006). The median $\hat {c}$ was estimated using 30 replicates at each design point. This process was repeated three times for the selected models and the results were respectively averaged. If $1\lt \hat {c}\lt 3$ then the model fits the data and the closer $\hat {c}$ is to 1, the better the fit. If $\hat {c}$ is outside of this range, the model does not fit and should be revised. Finally, estimates of the survival probability (*ϕ*) between each recapture event and the encounter probability (*p*) at each event were obtained by a weighted average of the best models and the overall *ϕ* and *p* were calculated by averaging pool estimates by habitat.

### Size and morphology

**Figure 1:**
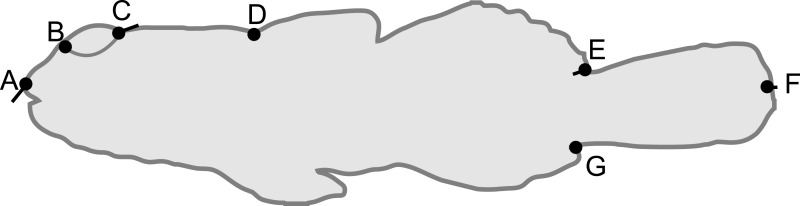
Schematic of *Bathygobius cocosensis* in side (lateral) view, illustrating the position of the seven landmarks (black filled circles) recorded to describe body shape. Landmarks were: tip of the snout (A), the anterior (B) and posterior (C) edges of the eye, the origin of the dorsal fin (D), dorsal caudal fin origin (E), the ventral origin of the caudal fin (F) and posterior point of caudal fin (G). Total length was measured as the distance between (A) and (F). Black vectors from landmarks illustrate the change in landmark position from low PC6 scores (short snout and small eyes) toward high PC6 scores (long snout and big eyes). Most of the change along this axis of phenotypic variation involved anterior-dorsal shift of landmark (A), and posterior shift in the position of landmark (C); some landmarks did not contribute variation to this component of shape variation. Vectors = 0.04 scale in MorphoJ.

Each captured *B. cocosensis* individual (once anaesthetised, see previous) was photographed using a NIKON Coolpix AW120 camera against a laminated graph paper background to provide uniform size scale; fish were photographed immediately after tagging, and any tagged fish that was re-captured was not photographed again, ensuring that our dataset does not contain repeated measures. Morphology was characterised by readily visible landmarks (Lele & Richtsmeier, 2001) (Fig. 1), localized on each photograph in IMAGEJ 1.48, after setting the scale using the background 1 mm grid (ImageJ, National Institutes of Health, Bethesda, Maryland, USA). Fish size was determined by total length (TL, distance between landmarks 1 and 6 in Fig. 1). In geometric morphometrics, centroid size, the square root of the sum of squared distances of each landmark to their centroid, is typically used to describe specimen size. Here, centroid size was highly correlated with TL (Pearson’s product moment correlation coefficient = 0.999, *d*.*f*. = 131, *P* < 0.0001), and we only analysed TL as it is readily comparable across studies.

A Generalized Procrustes Alignment (GPA), implemented in MorphoJ (Klingenberg, 2011), was used to align landmarks. The GPA removes scale (size), position and orientation (rotational) variation from the landmark coordinates (Bookstein, 1996; Adams, Rohlf & Slice, 2004; Zelditch, Swiderski & Sheets, 2012). This alignment generates collinearity among Procrustes aligned coordinates, reducing the dimensionality of the data from 2*p* (where *p* is the number of landmarks, which in our case was 7) to 2*p*-4 (Rohlf & Slice, 1990; Zelditch, Swiderski & Sheets, 2012). To remove this collinearity and to reduce the dimensionality of the data, a Principal Component Analysis (PCA) was conducted on the covariance matrix of the aligned landmark coordinates in MorphoJ (Klingenberg, 2011). The first seven (of 2*p*-4 = 10) Principal Components (PCs) explained 96.2% of the variance in body shape, and were retained for further analyses. All PCs were standardised (mean = 0 and standard deviation = 1) prior to analysis.

We also used Mahalanobis distance (De Maesschalck, Jouan-Rimbaud & Massart, 2000) to identify individuals that were multivariate outliers and outlier values were removed from the data, resulting in a total of seven missing records. Sample sizes were uneven among sites: there were a total of 41 low shore pool samples (19 and 22 from the two replicate pools) and 82 (35 and 47) high shore pool samples. If uneven sample sizes combine with unequal dispersion of samples, significance testing will be biased. The standard test for differences in variance between habitats is a Box’s *M* test, where a significance level of *P* ≤ 0.001 is generally taken as sufficient variance to bias sampling (Tabachnick & Fidell, 2007); in this instance the value indicated uneven variance (Box’s *M* = 48.02, *F*_28,23603.08_ = 1.59, *P* = 0.025), but insufficient to substantially bias analyses. Moreover, inspection of individual traits indicated that only PC1 differed in variance between habitats. When PC1 was excluded from the analysis (PCs 2–7 included), Box’s *M* no longer supported heterogeneous dispersion (Box’s *M* = 31.24, *F*_21,24735.10_ = 1.39, *P* = 0.107). Therefore to be conservative in our statistical tests, PC1 was excluded from the multivariate analysis.

Our experimental design corresponds to a mixed model nested ANOVA:

(1) \begin{eqnarray*}{y}_{ijk}=\mu +{\mathrm{Habitat}}_{i}+{\mathrm{Pool}}_{j(i)}+{\varepsilon }_{ijk}\end{eqnarray*}

where an individual’s trait value (*y*) was modelled as a function of the population mean (μ) and tide zone habitat (Habitat), which were fit as a fixed effects, and the replicate pool within each habitat (Pool) and the residual variance (ε), which were fit as a random effects. For body shape (PCs 2–6) the multivariate version of model Eq. (1) was fit, where *y* was a response vector of the six traits. Because there were only two replicate pools per habitat, we were unable to estimate an unconstrained covariance matrix for this random effect in the MANOVA. We therefore fit a diagonal covariance structure for this effect, estimating among pool variance for each trait, but not allowing covariance among traits. Using AIC we compared model fit of this covariance structure to more complex factor analytic covariance structures (Littell et al., 2006), which estimate reduced dimensions of variance. The diagonal covariance structure was the best-fit (smallest AIC), and therefore used in subsequent analyses.

Analyses were implemented using PROC MIXED in SAS (v. 9.4, SAS Institute Inc., 2012) under a maximum likelihood framework. To test for an effect of habitat on body shape (PCs 2–7) or size (TL), model 1 (above) was fit including or excluding the main effect of habitat, and a log-likelihood ratio test was used to determine if excluding habitat resulted in a significantly worse fit, with the degrees of freedom corresponding to the difference in the number of parameters fit (Wright, 1998). For the analysis of body shape, to determine which traits (PC2–PC6) contributed most to body shape differences between high and low tide pools, we fit habitat as a random effect, specifying a covariance structure with a single dimension as with only two levels of habitat (high and low tide pools) there can be only a single dimension of divergence between them. Specifically, we fit a first order factor analytic covariance structure (FA0(1): Littell et al., 2006) using restricted maximum likelihood. This analysis is analogous to a discriminant function analysis and identifies the trait combination that explained the variance between habitats when variation within habitats (among and within replicate pools) was appropriately taken into account.

## Results

### Site fidelity and survival probability

Site fidelity differed substantially with shore height for the four pools considered in our study (Table 1). A total of 222 individuals were tagged at least once, corresponding to 158 individuals collected in high shore pools and 64 individuals collected in low shore pools over the five-month study period. Fifty-three individuals were encountered in their original pool again (tagged twice minimally), corresponding to 40 individuals from high shore pools and 13 individuals from low shore pools (Table 2). The average density of *B. cocosensis* was somewhat greater in low pools (low pools, A: 0.088 ± 0.059; B: 0.010 ± 0.003; high pools, C: 0.032 ± 0.007; D: 0.033 ± 0.025 *B. cocosensis* per liter). Of the sixteen models for encounter probability (i.e., site fidelity) and survival, the two best models were indistinguishable based on AIC_*c*_ scores (299.74 and 300.39) with the remaining models having substantially poorer fits to the data (ΔAIC_*c*_ > 2; Table S1) and were not considered further. The two best models included: (i) encounter probability (*p*) that differed by group (i.e., high vs. low shore pools) and survival probability (*ϕ*) that did not differ by groups or time (best model: *ϕ*(.)*p*(*g*)) and, (ii) survival probability that differed by group and encounter probability that differed by group (second best model: *ϕ*(*g*)*p*(*g*)). Both models fitted the data well, with the average $\hat {c}=1.25$ for model (i) and $\hat {c}=1.19$ for model (ii). Estimates for the survival probability *ϕ* and the encounter probability *p* were obtained by averaging these two models together to obtain the most realistic estimates (Table S2). Thus, encounter probability in the high shore habitat was more than twice that in the low shore habitat (Table 1). This habitat-level difference in site fidelity was strongly supported by the inclusion of the *p*(*g*) term in both of the best-fit models. Survival was higher for both low shore pools than for the high shore pools, with average survival probability of low shore individuals 1.2 times that of high shore individuals (Table 1). Although this result is suggestive, the lack of difference in model fit between models (i) and (ii) indicates that the present data do not support a significant difference in survival between the habitats.

**Table 1:** Weighted average estimates of survival probability *ϕ* and encounter probability *p* per pool based on MARK models. Variables were estimated for each pool and the estimates were averaged by habitat.

Rockpool	Habitat	Survival probability *φ*	Encounter probability *p*
A	Low	0.739	0.131
B	Low	0.667	0.386
C	High	0.601	0.799
D	High	0.538	0.543
Averaged estimates	Low	0.703^a^	**0.259**^a^
	High	0.570^a^	**0.671**^a^

**Notes.**

a Averaged estimates by habitat are not significantly different for survival probabilities but are significantly different for encounter probabilities.

**Table 2:** Number of individuals newly captured and previously marked during each sampling event by habitat. For example, in October 55 fish were captured from high pools and of these 47 were new captures and eight had previously been marked. A total of 222 unique individuals were captured across the course of the experiment; of these, 53 were captured on multiple occasions with 10 recaptured more than twice.

	Low pools		High pools	
Month	New captures	Previously marked	New captures	Previously marked
Sept	17	*NA*	22	*NA*
Oct	5	2	47	8
Nov	9	3	16	21
Jan	10	5	23	7
Feb	23	3	50	17

**Figure 2:**
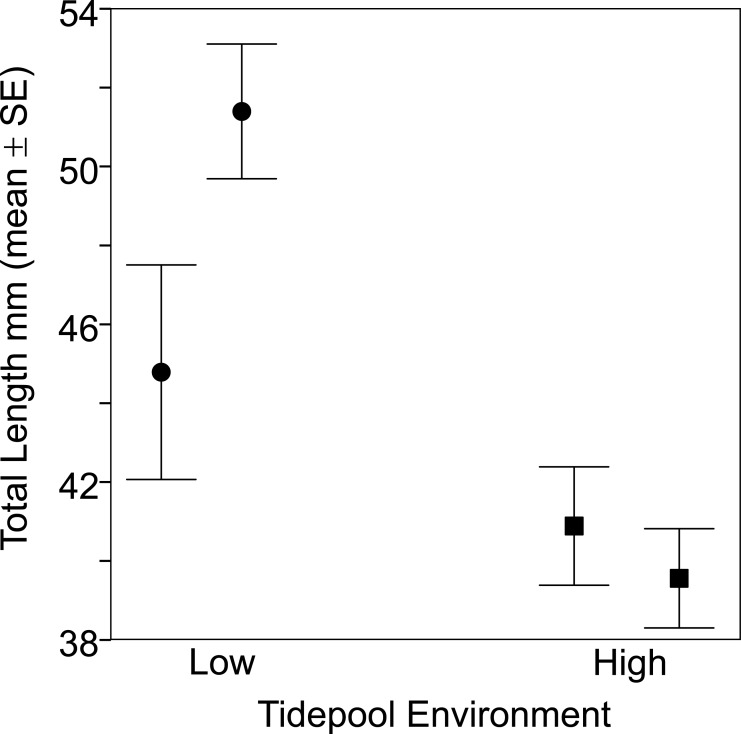
Fish from the two low tide pools (circles) were larger than those from the two high tide pools (squares) based on total body length (TL). Sample sizes per pool, left (low) to right (high), were 19, 22, 35 and 47, representing pools A–D respectively.

**Figure 3:**
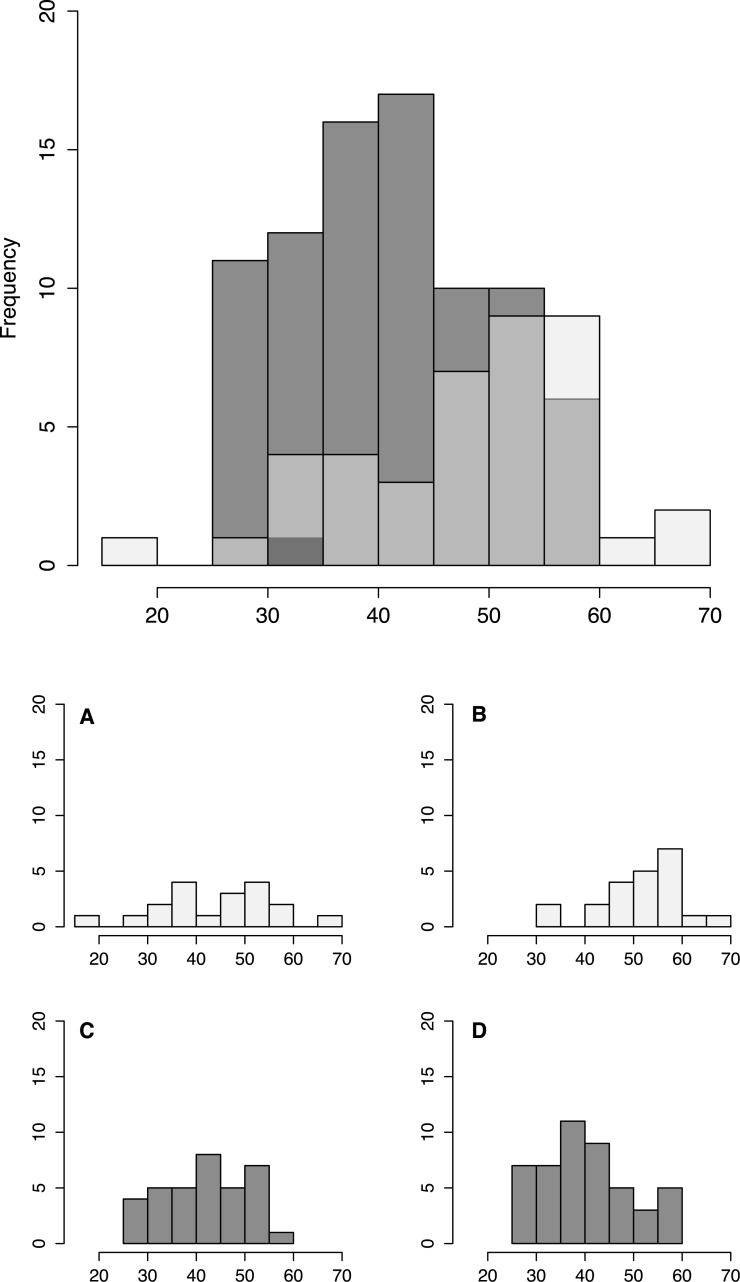
Fish size by tidepool. Size distribution of fish from low shore (white) and high shore (grey shaded) pools, with individual insets per pool (A–D).

### Size and morphology

Fish size and morphology differed significantly between high and low shore pools. Fish from the two low shore pools (mean TL = 48.25 mm ± 1.46 SE) were significantly bigger than fish from the two high shore pools (mean TL = 40.00 mm ± 1.00) (*χ*^2^ = 6.53, *d*.*f*. = 1, *P* = 0.011; Fig. 2). However, the size range within both habitats overlapped considerably, with fish captured from low shore pools spanning a wider range of sizes (18.6–68.3 mm) than fish from the high shore pools (25.4–59.9 mm) (Fig. 3). There appeared to be greater variance in size both between and within the two replicate low shore pools than for the replicate high shore pools (Figs. 2 and 3) but this pattern was not statistically supported (log-likelihood ratio test, LRT, comparing model with environment-specific between pool variance to a model with the same variance in both environments: *χ*^2^ = 1.26, *d*.*f*. = 1, *P* = 0.261).

**Figure 4:**
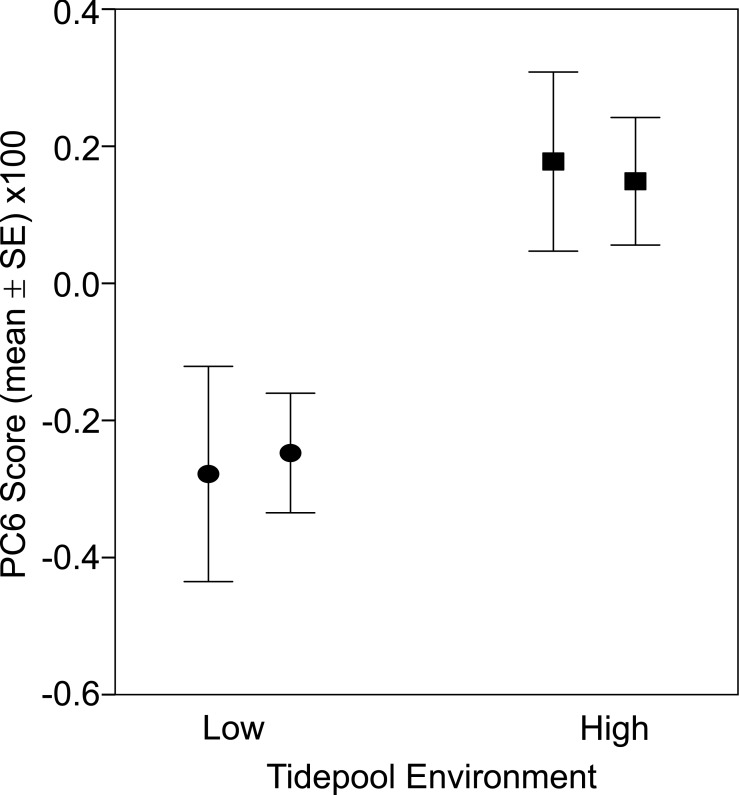
Fish from low (circles) and high (squares) tide pools differed in morphology as measured by PC6. The variation in body shape associated with PC6 is illustrated in Fig. 1, where snouts become longer and eyes larger as PC6 scores increase. Sample sizes per pool, left (low) to right (high), were 19, 22, 35 and 47, representing pools A–D respectively.

There was a significant difference in body shape between high and low shore pools (LRT of model with Habitat fit as a fixed effect vs. Habitat not fit in the model: *χ*^2^ = 16.07, *d*.*f*. = 6, *P* = 0.013). PC6 contributed most strongly to the body shape divergence between habitats (normalised vector loading 0.78). This axis of body shape variation was associated with variation in snout length and eye diameter (Fig. 1). On average, fish from the high tide zone pools had higher PC6 scores than did low shore pool individuals (Fig. 4), corresponding to longer snouts and larger eyes for their size. It is worth noting that PC6 explained only 3.38% of the total phenotypic variation across the dataset, suggesting that differences in habitat generated relatively subtle differences in morphology. To verify that PC6 was not affected by body size, we regressed PC6 on TL (not significant: *F*_1,121_ = 3.16, *R*^2^ = 1.7%, *P* = 0.078) and used thin plate spline exploration to check for a non-linear relationship, of which there was no evidence. For each trait (PCs 1–10), we used the mixed model to partition variation among habitats, among pools within habitats and to the residual variation among individuals within a pool (including measurement error). Over 96% of the total variance in body shape was attributed to variation within pools.

## Discussion

Although community structuring by shoreline height is a central principle in intertidal ecology, to our knowledge, our study is the first to suggest variation by shoreline height within an intertidal fish species for site fidelity, size, and morphology. For the resident goby *Bathygobius cocosensis*, we find that encounter probabilities are substantially greater in two high shore pools relative to two low shore pools consistent with higher site fidelity in the high intertidal. Size and morphological traits also differ by tidal position with high pool fish being overall smaller but presenting larger eyes and longer snouts for their size, and low pool fish being larger with proportionally smaller eyes and shorter snouts.

Previous studies have demonstrated strong site fidelity and homing behaviour in *B. cocosensis* (Griffiths, 2003a; White & Brown, 2013); our results match these findings but also uncover differences between high and low shore fish. From our four pools, we recaptured 55 of 222 tagged fish over our study period of 150 days (23% recaptured: Table 1). These results are in striking agreement with those from a single rockpool sampled by Griffiths (2003a) where 11 of 46 tagged fish (24%) were recovered from the same pool over the study duration of 102 days; White & Brown (2013) had higher recovery rates, 77%, but a shorter time (42 days). Because neither of these previous studies used a repeat sampling design, it is not possible to segregate losses due to emigration or mortality. Our analyses and interpretation of site fidelity and mortality are based on the CJS model (Cormack, 1964; Jolly, 1965; Seber, 1965), which provides modelled estimates of encounter probability (i.e., site fidelity) and survivorship.

In comparing high and low intertidal rockpools, we found that there was stronger site fidelity of *B. cocosensis* in the high intertidal pools with an overall encounter probability of 67% (i.e., 67% of sampled individuals who were still living at the next monthly sampling increment were expected to be re-encountered in the same pool). In contrast, low shore pool individuals presented only a 26% probability of being re-encountered (Table 1). These findings match expectations: high shore fish would have few opportunities relative to low shore fish to forage outside of their home pools, as these pools are landlocked for most of the tidal cycle. In addition, should high shore fish be unable to return to their home pool they would be challenged to find suitable pools able to sustain them for long periods until the next tidal flooding and thus could be stranded in unsuitable areas and die (Williams, 1957; Gibson, 1972; Yoshiyama et al., 1992; White & Brown, 2013). In contrast, low shore individuals experience shorter isolation times and the possibilities to change pools are greater as much of the habitat is suitable on the low shore line (Griffiths, 2003a). In addition, the high density of both *B. cocosensis* and total fishes in the low intertidal may increase pressure to emigrate out of high density pools.

Accompanying the substantial differences in recapture probability (site fidelity), the estimated survival probability of individuals may differ between low and high shore pools. Our data indicate a trend of lower survival probability in high pool individuals (Table 1). Although *B. cocosensis* are well adapted to environmental variation, stranding in unsuitable pools during low tide may increase chances of mortality (Williams, 1957; Yoshiyama et al., 1992). Given that fish predation and interspecific competition are relatively low in the high intertidal zone (Menge & Sutherland, 1987) and that this species is well camouflaged (Gibson, 1999; Griffiths, 2003b) and, thus, unlikely to be strongly affected by bird predation and human nuisance, the high physiological pressures of the high intertidal zone are the most likely source of possible differential selection. Our data are suggestive of higher mortality in the high intertidal, but the statistical support for differential mortality was non-significant, so with the limited data in hand no conclusions are possible.

The CJS model underlying our estimates of site fidelity and survivorship relies on repeat observations of tagged and untagged individuals, where untagged individuals are assumed to be immigrants to the pool. Although VIE tags are generally well retained (Catalano et al., 2001; Olsen & Vøllestad, 2001; Josephson et al., 2008) and a previous examination of tag retention in *B. cocosensis* found high tag retention for fish held in aquaria (Griffiths, 2002; White & Brown, 2013), we cannot know the rates of tag loss in the wild. Similarly, although we did not directly observe mortality associated with our handling of live fish, we cannot exclude some post-release mortality resulting from tagging. However, unless tag loss varied by habitat, this missing parameter should not bias our main conclusions of a difference in site fidelity between habitats. Differential tag shedding by habitat seems unlikely and therefore the detected difference in site fidelity among habitats should be free from bias arising from tagging effects. A habitat by tag-induced mortality effect is possible (for instance, the higher mortality in high tide pools could reflect greater sensitivity of post-anaesthesia fish to physiological stressors or enhanced predation risk), but no significant differences in mortality between habitats were detected (see above).

Fish were larger on average in our low shore pools (Fig. 2). This result contrasts with that of Cox et al. (2011) who found no difference in body size of *B. cocosensis* across low, mid and high shoreline pools on O’ahu. This difference might reflect inter-population variation of the effect of vertical positioning within* B. cocosensis*. In some species, larger individual gobies have been shown to be better competitors for resources (Magnhagen & Kvarnemo, 1989), and larger individuals could be competitively excluding small individuals from low shore pools. An alternative but not mutually exclusive mechanism generating variation in body size could be the higher metabolic cost of large individuals in warm temperatures (Hernández et al., 2002). Thus larger individuals are expected to have lower fitness than smaller individuals in the high intertidal zone (Hernández et al., 2002; White, Hose & Brown, 2015). Notably, we found most size classes to be present in all pools (25–60 mm TL: Fig. 3), indicating that small fish were not definitively excluded from the low tide pools and larger adults were able to occupy high tide pools. Further work is required to determine whether smaller fish present in the upper intertidal are young fish, still growing, or whether other factors (e.g., physiological stress, low food abundance) contribute to their small size.

This study was the first to investigate morphological variation within fish species with respect to intertidal zonation. Overall, very little of the variation in body shape was distributed between low and high tide habitats; rather most of the variation in body shape was attributable to differences among individuals within a pool. Nonetheless, we found statistically significant morphological differences between high and low shore fish (Fig. 4), consistent with *B. cocosensis* experiencing distinct environments in the high and low intertidal zones. Fish from the high tide zone had longer snouts and bigger eyes for their size. Although subtle, these statistically significant differences in morphology by habitat are intriguing and a novel observation.

The morphological disparity in fishes distributed across habitats could be generated by several, not mutually exclusive, mechanisms that we cannot resolve fully with the current data. If morphologically different individuals choose to reside in different habitats (environmental matching) the morphological differences will arise irrespective of mechanisms underlying pool choice. In particular, differential habitat choice by fish of different ages or life history stages could have generated our observed pattern of morphological variation if there are both allometric changes and different habitat usages by size. Yet, there was no statistical support for the shape variation captured by PC6 changing with fish size (*R*^2^ = 1.7%, *P* = 0.078 and thin plate spline exploration revealed no indication of a non-linear relationship), suggesting that morphological differences are not due to simple allometric change and differences in fish size (age) across habitats. Habitat matching not related to body size, nonetheless, remains a possibility. Morphological differentiation could alternatively be generated by phenotypic plasticity (i.e., variation induced between genetically homogenous individuals by the environment they experience) (Stearns, 1989; West-Eberhard, 1989; Faulks et al., 2015) in direct response to biotic or abiotic differences between low and high tide pools. Viability selection for certain morphological features, resulting in differential mortality of fish, could also result in the observed morphology—habitat association. Our observation of morphological differentiation across micro-environmental scales (within the dispersal neighbourhood) is fundamentally similar to findings in amphidromous gobies (Moody et al., 2015) and leaping blennies (Morgans, Cooke & Ord, 2014) where populations show significant morphological differences. Whether divergent selection could cause morphological differences across the intertidal is not known. In summary, phenotypic plasticity or habitat matching (with or without post-settlement selection) are the most likely mechanisms for morphological disparities between high and low rockpool *B. cocosensis.*

For *B. cocosensis*, variance in morphology by shore pool height could be a result of prey availability differences in these habitats, leading to diet differences and morphological responses due to ontogenetic niche shifts, phenotypic plasticity, habitat matching or selection. In other fishes, diet has been shown to affect head shape, including eye diameter, snout length, head width and jaw length (Meyer, 1987; Witte, Barel & Hoogerhoud, 1989; Wimberger, 1992; Day, Pritchard & Schluter, 1994; Hegrenes, 2001), and habitat-induced allometries between eye and total body size have also been noted (Pankhurst, 1992; Pankhurst & Montgomery, 1994). *B. cocosensis* feed on small benthic invertebrates (Griffiths, 2003a; Cox et al., 2011); however, their full spectrum of prey has never been detailed. Therefore, is it conceivable that, depending on the microhabitat individuals occupy, dietary differences exist, and underlie the observed divergence in head shape.

## Conclusions

The findings of significant differences in site fidelity, body size, and morphology support the conjecture that ecological variation, even on micro spatial scales, generates intra-specific variation within *B. cocosensis*. Patterns of survival probability, although not statistically significant, also support this conclusion. This study demonstrated that population dynamics in the intertidal environment are complex and require the investigation of many factors to increase understanding of this environment. Future work will seek to determine whether the observed patterns are consistent with greater numbers of pools sampled per habitat, persist over longer time periods, and are general across different locations. The presence of morphological variation along intertidal gradients has not been previously observed among fish species and provides a foundation for further studies on ontogeny, phenotypic plasticity and natural selection.

##  Supplemental Information

10.7717/peerj.2263/supp-1 Supplemental Information 1 Information for Encounter History dataset

10.7717/peerj.2263/supp-2 Supplemental Information 2 Information for lateral morphology dataset

10.7717/peerj.2263/supp-3 Supplemental Information 3 Encounter history dataset infile for MARK software

10.7717/peerj.2263/supp-4 Supplemental Information 4 Lateral morphology dataset R ready csv format

10.7717/peerj.2263/supp-5 Figure S1 Aerial view of the tidal platform and sampling pools at Hastings Point, NSW

10.7717/peerj.2263/supp-6 Figure S2 Typical high (1 & 2) and low (3 & 4) pools. Photo #2 is that of sampled pool B

10.7717/peerj.2263/supp-7 Supplemental Information 5 Tables S1 & S2

## Additional Information and Declarations

### Field Study Permissions

The following information was supplied relating to field study approvals (i.e., approving body and any reference numbers): Fish were collected under NSW Department of Primary Industries Scientific Collection Permit P13/0046-1.

### Data Availability

The following information was supplied regarding data availability: The raw data has been supplied as Supplemental Dataset.
