# Supplementary material for: Site fidelity, size, and morphology may differ by tidal position for an intertidal fish, Bathygobius cocosensis (Perciformes-Gobiidae), in Eastern Australia
Source: PeerJ. 2016 Jul 28;4:e2263. doi: 10.7717/peerj.2263 (PMC4974941; doi:10.7717/peerj.2263)
Supplement: Figure S1 [file peerj-04-2263-s005.pdf]

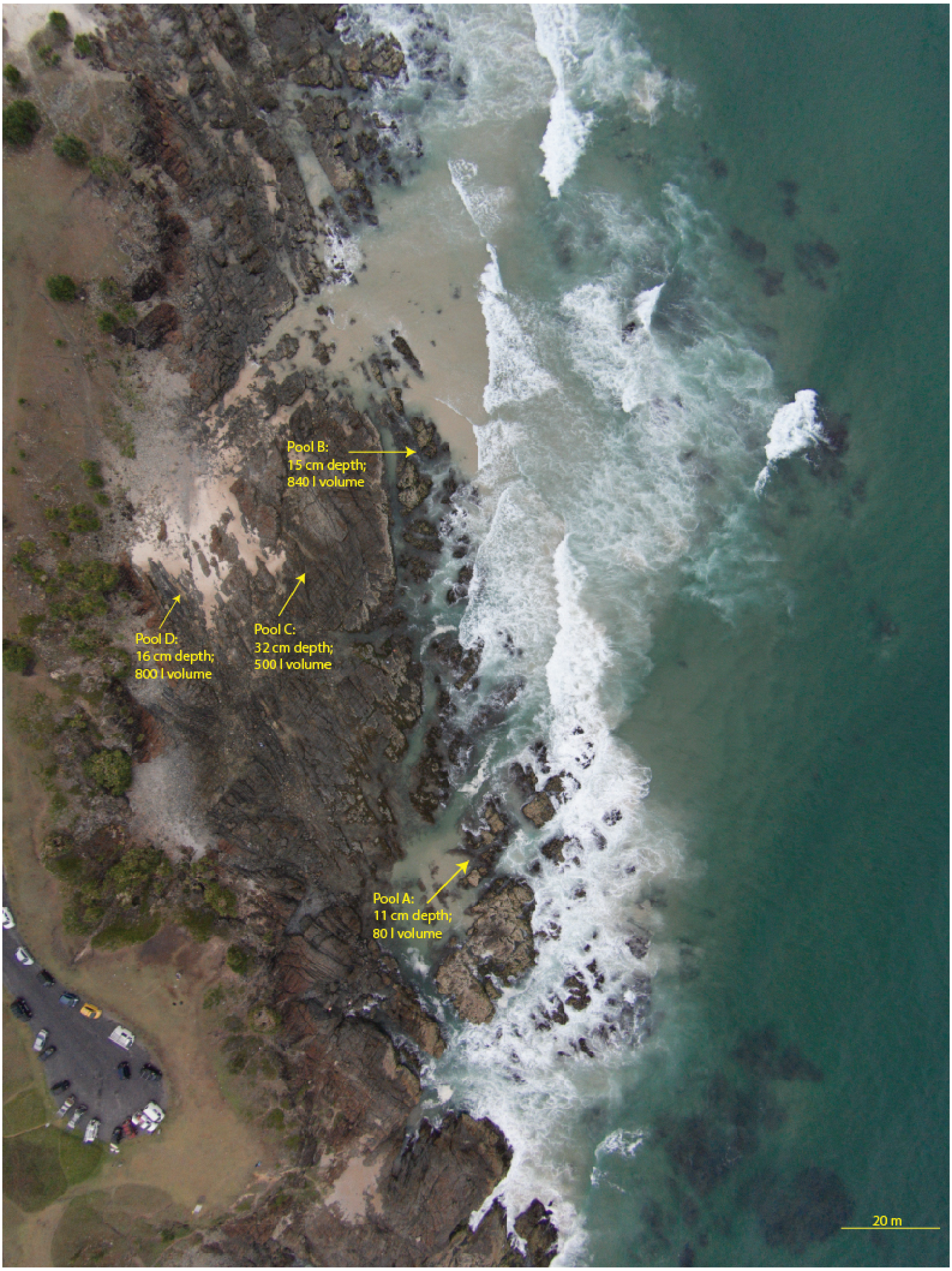

Supplemental Figure 1: Aerial view of the tidal platform and sampling pools at Hastings Point, NSW.
