# Supplementary figures and images for: Site fidelity, size, and morphology may differ by tidal position for an intertidal fish, Bathygobius cocosensis (Perciformes-Gobiidae), in Eastern Australia
Source: PeerJ. 2016 Jul 28;4:e2263. doi: 10.7717/peerj.2263 (PMC4974941; doi:10.7717/peerj.2263)

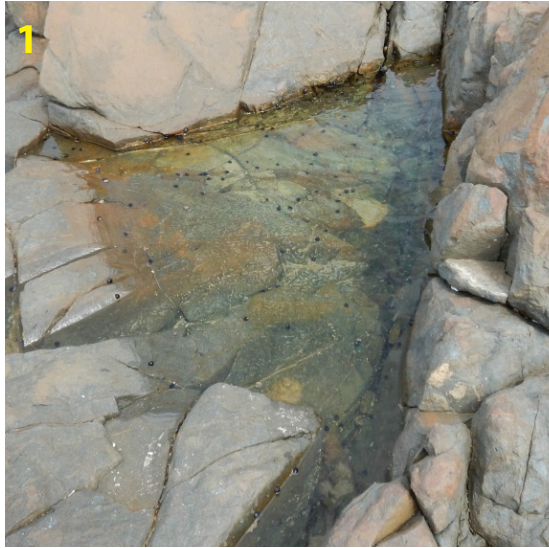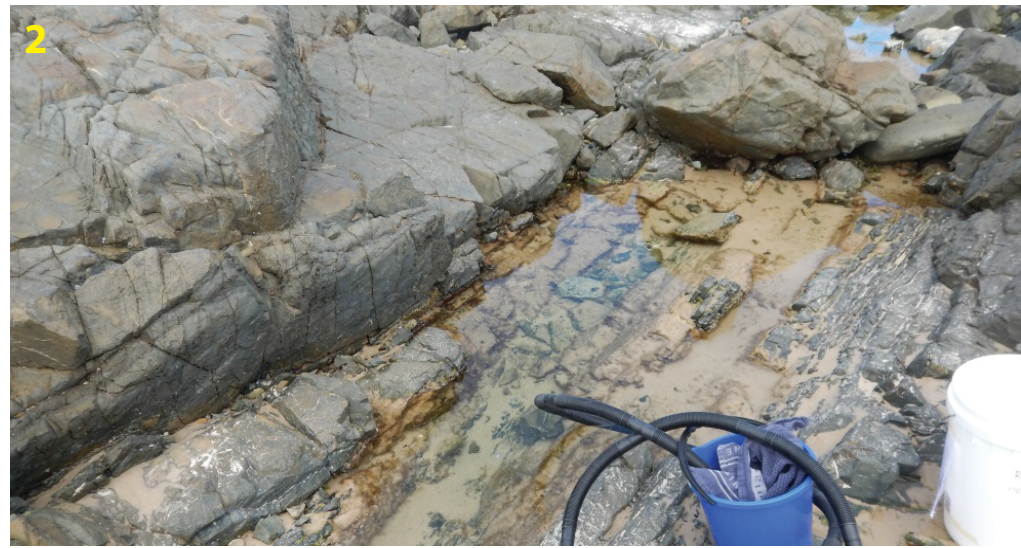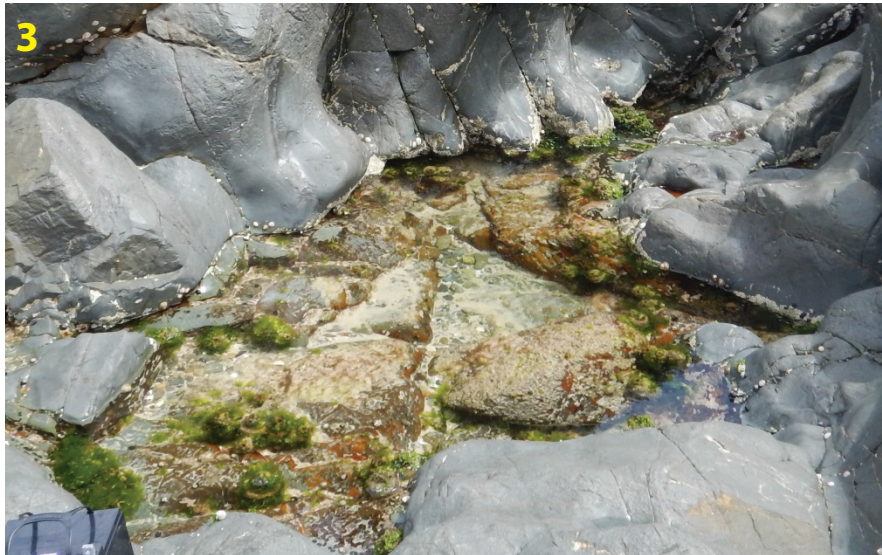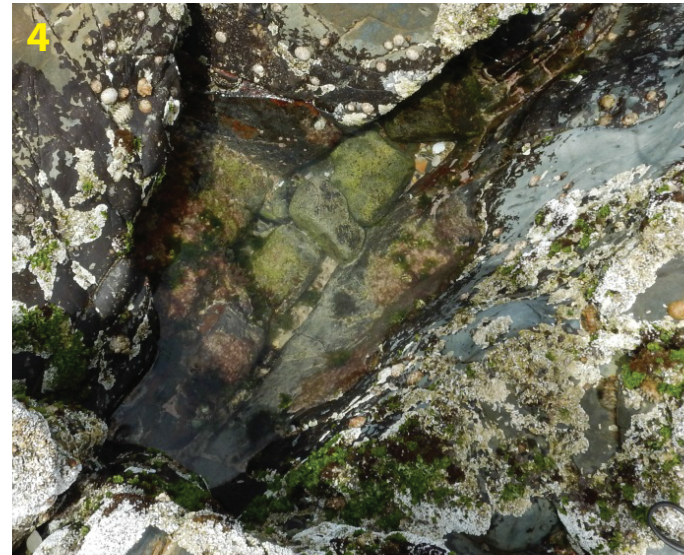

Supplemental Figure 2: Typical high (1 & 2) and low (3 & 4) pools. Photo #2 is that of sampled pool B.

Supplement: Figure S2 [file peerj-04-2263-s006.pdf]
