# Supplementary material for: Site fidelity, size, and morphology may differ by tidal position for an intertidal fish, Bathygobius cocosensis (Perciformes-Gobiidae), in Eastern Australia
Source: PeerJ. 2016 Jul 28;4:e2263. doi: 10.7717/peerj.2263 (PMC4974941; doi:10.7717/peerj.2263)
Supplement: Supplemental Information 5 [file peerj-04-2263-s007.pdf]

SUPPLEMENTARY MATERIALS

Table 1. Support for specific models of survival and site fidelity from MARK. MARK models are ordered by AIC_c values. The best two models indicate that encounter probability differs by habitat grouping (*p(g)*).

**Model**[a]	AIC_c [b]	ΔAIC_c [b]	w_i [b]
Φ (.) p(g)	299.744	0.00	0.53797
Φ (g) p(g)	300.393	0.65	0.38883
Φ (t) p(g)	304.885	5.14	0.04115
Φ (.) p(.)	307.904	8.16	0.00910
Φ (g) p(.)	308.082	8.34	0.00832
Φ (.) p(g*t)	308.944	9.20	0.00541
Φ (g) p(g*t)	309.656	9.91	0.00379
Φ (g*t) p(g)	311.440	11.70	0.00155
Φ (g) p(t)	312.183	12.44	0.00107
Φ (.) p(t)	312.501	12.76	0.00091
Φ (t) p(.)	313.327	13.58	0.00060
Φ (t) p(g*t)	313.529	13.79	0.00055
Φ (t) p(t)	314.347	14.60	0.00036
Φ (g*t) p(.)	314.775	15.03	0.00029
Φ (g*t) p(t)	317.959	18.22	0.00006
Φ (g*t) p(g*t)	318.993	19.25	0.00004

[a] Φ is the survival probability and p is the encounter probability; full stops (.) indicate a single value for both habitats; (g) indicates that value differ by habitat group; (t) indicates that values differ by time; and asterisks (*g*t*) indicate an interaction term between group and time.

[b] The lowest AIC_c indicates the model fitting mark-recapture data best; ΔAIC_c is the AIC_c difference between the best and individual model; w_i is the support weight for each model.

Table 2. Details of model averaging for to yield rockpool specific estimates of survival (*Φ*) and encounter probability (*p*). Variable specific MARK estimates for the best two models are reported and a weighted average of models was conducted as recommended in the MARK guidelines (Cooch and White 2006).

			Φ		*p*	
**Rockpool ID**	**Model**	**weight**	**estimate**	**SE**	**estimate**	**SE**
A	*Φ(.) p(g)*	0.580	0.596	0.048	0.172	0.099
	Φ(g) p(g)	0.420	0.936	0.335	0.076	0.078
	Weighted Average		0.739	0.168	0.131	0.090
B	*Φ(.) p(g)*	0.580	0.596	0.048	0.441	0.133
	Φ(g) p(g)	0.420	0.766	0.138	0.309	0.142
	Weighted Average		0.667	0.086	0.386	0.137
C	*Φ(.) p(g)*	0.580	0.596	0.048	0.801	0.095
	Φ(g) p(g)	0.420	0.607	0.063	0.796	0.100
	Weighted Average		0.601	0.054	0.799	0.097
D	*Φ(.) p(g)*	0.580	0.596	0.048	0.468	0.115
	Φ(g) p(g)	0.420	0.457	0.078	0.648	0.154
	Weighted Average		0.538	0.060	0.543	0.131
